# Navigation programs to support community-dwelling individuals with life-limiting illness: determinants of implementation

**DOI:** 10.1186/s12913-024-10541-y

**Published:** 2024-01-06

**Authors:** Sarah Scruton, Grace Warner, Cynthia Kendell, Kathryn Pfaff, Kelli Stajduhar, Linda Patrick, Carren Dujela, Faith Fauteux, Robin Urquhart

**Affiliations:** 1 Department of Community Health and Epidemiology, Centre for Clinical Research, Dalhousie University, Room 413, 5790 University Avenue, Halifax, NS B3H 1V7 Canada; 2 School of Occupational Therapy, Dalhousie University, Halifax, NS Canada; 3 Department of Medicine, Dalhousie University, Halifax, NS Canada; 4 Faculty of Nursing, University of Windsor, Windsor, ON Canada; 5 School of Nursing, University of Victoria, Victoria, BC Canada

**Keywords:** Implementation science, End-of-life care, Navigation programs, Community care, Chronic disease

## Abstract

**Background:**

As the Canadian population ages and the prevalence of chronic illnesses increases, delivering high-quality care to individuals with advanced life limiting illnesses becomes more challenging. Community-based navigation programs are a promising approach to address these challenges, but little is known about how these programs are successfully implemented to meet the needs of this population. This study sought to identify the key determinants that contribute to the successful implementation of these programs within Canada.

**Methods:**

A qualitative study was undertaken to understand the implementation of eleven innovative, community-based navigation programs that aim to address the needs of individuals with life-limiting illnesses as they approach the end of life. The Consolidated Framework for Implementation Research (CFIR) guided the study design. Key informants (*n* = 23) within these programs took part in semi-structured interviews where they were asked to discuss how these programs are implemented. Data were analyzed using techniques employed in qualitative description.

**Results:**

We identified key determinants of successful implementation within each CFIR domain. In the outer setting domain, participants emphasized the importance of filling gaps in care to meet client needs, developing strong relationships with clients and community-based organizations, and navigating relationships with healthcare providers. At the inner setting level, leadership support, staff compatibility, and available resources were identified as important factors. In terms of intervention characteristics, the ability to adapt was cited as a facilitator, whereas costs were identified as a barrier. For the characteristics of individuals, participants described the importance of having staff whose values align with the program, and who have the experience and skills necessary to work with complex clients. Finally, having strong champions and evaluation processes were highlighted as important process-oriented determinants of successful implementation.

**Conclusion:**

This study provides valuable insights into the determinants of successful implementation of community-based navigation programs in Canada. Understanding these determinants can guide the future development and integration of navigation programs to successfully meet the needs of those with life-limiting illnesses.

**Supplementary Information:**

The online version contains supplementary material available at 10.1186/s12913-024-10541-y.

## Introduction

As the proportion of Canadians over the age of 65 continues to grow [1], and the prevalence of chronic illness increases [2], it becomes more challenging to deliver high-quality care to individuals with advanced life-limiting illnesses who are nearing the end of life (EOL). Numerous studies within Canada have shown that the current healthcare system often fails to meet the needs of these individuals [3–6]. For example, although many people who are nearing the EOL would rather remain and die at home, EOL care is mainly delivered in hospitals and is not well-coordinated [7–9]. In order for these individuals to remain in the community instead, appropriate supports are required. However, patients, caregivers, and healthcare providers are often unaware of the community supports that are available to assist individuals with life-limiting illnesses as an alternative to in-hospital care [10–12]. Moreover, the numerous providers, services, and referral pathways can be confusing and difficult to navigate [13–16].

One promising approach to addressing these challenges is the delivery of community-based navigation programs, which have been successful at educating patients and their families, connecting them to critical health system and community supports, and enabling coordination across healthcare settings [17–23]. Though less commonly studied, navigation programs aimed at improving EOL outcomes for patients and their families have been shown effective [24–26]. These programs include a wide range of models and interventions, such as those led by multi-disciplinary teams, nurses, or volunteers. Key components of these programs typically involve advance care planning, patient education, symptom management, and connection to or leverage of community resources. Multiple studies within Canada have demonstrated beneficial outcomes (e.g., patient satisfaction, knowledge of available resources, remaining in their preferred setting at the time of death) in nurse-led [24] and volunteer-led [27] models. A comprehensive global review examining the effectiveness of in-home EOL care programs by Bainbridge et al. reinforces the consistency of these findings worldwide, as 40 different interventions demonstrated improvements in patient outcomes such as quality of life, satisfaction with care, pain management, and cost savings [25]. Several teams within Canada have implemented these programs to improve the quality of life of patients and their families (patients and families will hereafter be referred to as clients) facing EOL [24, 27, 28].

Despite their benefits for clients, limited research has sought to understand how these programs are implemented into practice, and the facilitators and barriers that influence their implementation. This knowledge gap results in a critical challenge for the development and implementation of future navigation programs. Researchers have begun to explore implementation processes for navigation programs in other care areas, with strong partnerships across stakeholder groups, leadership support, funding stability, and adequate staff support all determinants of program implementation and success [29, 30]. In this study, the research team sought to address two objectives: (1) to examine how navigation programs address the needs of those living in the community with advanced, life-limiting illness, and (2) to identify the determinants of successful implementation of these programs. The findings relevant to the first objective are reported elsewhere [31]. In summary, our previous study found that these programs successfully meet the needs of their clients when staff are trained, supported, and empowered to take the time needed to personalize care to their clients’ needs and circumstances [31].

This paper focuses on the work that was undertaken relevant to the second objective (i.e., determinants of implementation). The specific objective was to explore the key determinants (facilitators and barriers) that contributed to the successful implementation of innovative, community-based navigation programs in Canada, from the perspective of the individuals who are involved in delivering them.

## Methods

### Study design

We used qualitative inquiry, informed by realist evaluation methods [32], to address the study objectives. Specifically, we conducted semi-structured interviews with staff of innovative navigation programs across Canadian provinces that deliver services to individuals affected by life-limiting illness, and their families. Approval to conduct the study was received from the Nova Scotia Health Research Ethics Board, the University of Victoria Research Ethics Board, and the University of Windsor Research Ethics Board.

### Conceptual approach

To explore the implementation of these programs in greater depth, we used the Consolidated Framework for Implementation Research (CFIR) [33]. CFIR is a conceptual framework that assimilates constructs from a number of influential frameworks and models in the knowledge translation/implementation science literature and thus provides an approach to studying and understanding the multiple levels that influence implementation processes. The framework includes 5 domains that influence implementation, including: the intervention (e.g., adaptability, costs), outer setting (e.g., resources, client needs), inner setting (e.g., culture, structural characteristics), individuals involved (e.g., staff knowledge, identification with intervention), and implementation process (e.g., engagement, evaluation) [33]. This framework was used to inform the interview guide and analyses, as the findings were sorted and categorized based on these five domains and the examples provided in the CFIR.

### Data collection

To identify community-based navigation programs, we conducted a horizon scan using an adapted Agency for Healthcare Research & Quality approach [34]. Our focus was on identifying innovative, provincial level programs that aimed to address the needs of community-dwelling clients who are experiencing advanced life-limiting illnesses as they approach EOL. The identification of programs is reported in detail elsewhere [31]. In summary, the programs were identified from websites, grey literature, and a nomination process involving direct contact with research, clinical, and policy leaders in each of the 10 Canadian provinces. The final selection of eligible programs was based on conversations with the research team to ensure each included program incorporated the specific criteria detailed in Table 1 [35, 36].

**Table 1:** Eligibility criteria for navigation programs

1. The program serves individuals living in their community with chronic, life-limiting illness.
2. The program is located in either a hospital or community setting.
3. The program includes dedicated staff or volunteers who provide navigation services (may be referred to as “Navigators”). As described by Valaitis et al, navigation services include: patient education, care planning, home visits, promoting coordination and continuity across health settings, and early identification of and response to health changes [36].
4. The program must be affiliated with a host organization and have some sort of governance structure and accountability.
5. The program delivers services consistent with a palliative approach to care. This is described by Touzal and Shadd: “a palliative approach exists when care simultaneously addresses whole-person needs, enhances quality of life, and acknowledges mortality. This model is applicable to care provided in any setting, by any provider, to any patient with a life-threatening illness, at any point in the illness trajectory” [35].

Semi-structured interviews were conducted with key informants who were familiar with how the program worked from an operational structure and organizational perspective. This included individuals responsible for the management and delivery of the programs, and decision-makers responsible for overseeing the programs. After being identified as an eligible program, a research coordinator (CK, FF, or CD) contacted potential participants via e-mail to determine their willingness to take part in the study. If they expressed interest, the research coordinator initiated the informed consent process and scheduled an interview.

Research coordinators with experience in qualitative methods (CK, FF, CD) carried out the interviews, which were in-person or via telephone or videoconferencing, depending on the participant’s preference. Interviews focused on four main topics: (1) how the program operated (e.g., structure, services provided, key personnel), (2) impacts of the program for clients, (3) how the program was integrated with/interacted with the health care system and the community, and (4) implementation and sustainability. This analysis focused on the fourth topic regarding responses to questions about implementation and sustainability, whereas the other three topics are discussed in a previously published study focused on understanding how these programs function to meet the needs of their clients [31]. The interview guide was open-ended and adapted as needed, based on the role of the individual being interviewed (see Additional File 1). Interview duration ranged from 24:56 to 1:28:40. The interviews were audio-recorded and transcribed. No repeat interviews were conducted.

### Data analysis

Members of the research team (RU, GW, KS, KP, LP, CK, FF, CD) read and re-read the transcripts from the semi-structured interviews, and developed initial program theories, as aligned with realist evaluation. Next, a research assistant (SS) coded the transcripts employing qualitative descriptive methods [37] and using NVivo 12 to identify the contexts, mechanisms, and outcomes that explain how these programs work as well as the determinants of successful implementation (the focus of this inquiry). Coding entailed the reading and rereading of transcripts, recording insights and reflections on the data, and sorting the data to identify similar concepts, patterns, and important features [37]. The coding was reviewed by team members RU, GW, and CK. Through this process, determinants of implementation were identified, and then iteratively discussed and refined by the research team. The final determinants were organized and presented according to the five CFIR domains.

## Results

Twenty-three participants were interviewed from 11 navigation programs from across Canada. These programs operated in five Canadian provinces (Nova Scotia, Ontario, Alberta, British Columbia, and Prince Edward Island) and represented a mixture of community (non-profit or volunteer) and health system programs. While all programs supported people with life-limiting illness in their communities, some were positioned in hospital (outpatient) settings, some were positioned in, or closely connected to, standalone hospices, some were positioned in non-profit community-based organizations, and some were positioned in academic institutions (i.e., those programs that were research-based). Only two were funded through the health system; all remaining programs were funded via government grants, research grants, and/or donors and community fundraising. The individuals who delivered navigation services included highly trained healthcare providers (e.g., physicians, nurses, and allied health professionals), client services/program staff, trained volunteers, and persons with lived caregiving experience. Only four programs did not provide home visits to clients, but these programs did provide virtual and telephone-based appointments so clients did not have to leave their homes to avail of their services. Tables 2 and 3 describe the programs in terms of these characteristics. In each program, we interviewed frontline staff/volunteers who deliver navigational services and individuals in leadership (decision-making) roles.

**Table 2:** Description of programs (A-F) by key contextual characteristics. Programs are anonymized by letter

Program	A	B	C	D	E	F
**Setting**	Community	Hospital^a^	Community	Hospital	Academic	Academic
**Funding**	Non-profit	Health system	Non-profit	Health system	Non-profit	Research grants
**Navigator type**	Program staff^b^	Multidisciplinary healthcare team	Program staff	Multidisciplinary healthcare team	Volunteer	Volunteer
**Home visits**	Yes	Yes	No	Yes	No	Yes

^a^Hospital based programs were ambulatory (outpatient) programs, not inpatient programs

^b^Paid staff of the program, who are in navigator roles but who are not healthcare professionals

**Table 3:** Description of programs (G-K) by key contextual characteristics. Programs are anonymized by letter

Program	G	H	I	J	K
**Setting**	Community	Academic	Community	Community	Hospice
**Funding**	Government	Research grants	Non-profit	Non-profit	Non-profit
**Navigator type**	Program staff*	Volunteer	Persons with lived experience	Persons with lived experience	Volunteers
**Home visits**	Yes	Yes	No	No	Yes

^*^Paid staff of the program, who are in navigator roles but who are not healthcare professionals

Key determinants to implementation existed within all CFIR domains. Table 4 presents the main themes (determinants) in relation to the CFIR domains and constructs. Table 5 presents the determinants, and whether each was a facilitator and/or barrier, for each program. Although not tied to initial implementation efforts, for some programs, scale-up (i.e., expansion of the program to new locations or populations) was a high priority and viewed as a part of their long-term implementation strategy. These were programs that had undergone multiple and/or ongoing evaluations (research-oriented or otherwise) and had demonstrated value in terms of patient and health system benefits. Therefore, where relevant to the program/participants, the concept of scale-up is discussed in the domains below.

**Table 4:** Themes as they relate to CFIR domains and constructs

CFIR Domain	Theme (Corresponding CFIR Construct)
Outer Setting	Responding to client needs facilitates implementation (Patient needs and resources)
	Strong inter-organizational relationships facilitate implementation (Cosmopolitism)
	Limited awareness of program impedes implementation (No direct CFIR construct)
Inner Setting	Leadership support and engagement facilitate implementation (Leadership engagement)
	Fit between staff values and skills and the program’s philosophy facilitates implementation (Compatibility)
	Resource availability (or lack thereof) facilitate or impede implementation (Available resources)
Intervention Characteristics	Ability to adapt program components and delivery facilitates implementation (Adaptability)
	Inadequate funding to deliver the program impedes implementation (Cost)
Process of Implementation	Champions facilitate implementation (Champions)
	Evaluation facilitates implementation, refinement, and scale-up (Reflecting and evaluating)
Characteristics of Individuals	Team members’ knowledge and attributes facilitate implementation (Personal attributes)

**Table 5:** Determinants, and whether each was a facilitator and/or barrier, for each program. + indicates the determinant was a facilitator; – indicates the determinant was a barrier; ± indicates the determinant was both a facilitator and barrier; n/a indicates the determinant was not described by participants as being particularly relevant to the program’s implementation

Program	A	B	C	D	E	F	G	H	I	J	K
Outer Setting											
Responding to client needs facilitates implementation (*Patient needs and resources*)	+	+	+	+	+	+	+	+	+	+	+
Strong inter-organizational relationships facilitate implementation (*Cosmopolitism*)	+	+	+	+	n/a	±	±	+	±	+	+
Limited awareness of program impedes implementation (*No direct CFIR construct*)	-	n/a	n/a	n/a	n/a	n/a	-	-	-	-	-
Inner Setting											
Leadership support and engagement facilitate implementation (*Leadership engagement*)	n/a	+	+	n/a	n/a	+	n/a	+	n/a	n/a	+
Fit between staff values and skills and the program’s philosophy facilitates implementation (*Compatibility*)	+	+	n/a	n/a	+	+	+	n/a	+	+	n/a
Resource availability (or lack thereof) facilitate or impede implementation (*Available resources*)	-	±	+	±	n/a	+	+	+	+	+	-
Intervention Characteristics											
Ability to adapt program components and delivery facilitates implementation (*Adaptability*)	+	+	n/a	+	n/a	+	n/a	+	+	+	+
Inadequate funding to deliver the program impedes implementation (*Cost*)	-	-	-	n/a	-	n/a	-	n/a	n/a	-	-
Process of implementation											
Champions facilitate implementation (*Champions*)	+	+	n/a	n/a	n/a	+	n/a	+	n/a	n/a	+
Evaluation facilitates implementation, refinement, and scale-up (*Reflecting and evaluating*)	+	±	n/a	±	n/a	+	+	+	+	+	+
Characteristics of Individuals											
Team members’ knowledge and attributes facilitate implementation (*Personal attributes*)	+	+	+	+	+	+	+	+	+	+	+

### Outer setting

Participants described three determinants of implementation that were particularly relevant to the outer setting: client needs and relationships, inter-organizational relationships, and awareness of the program.

*Responding to client needs facilitates implementation.* Across programs, participants described the emergence of their navigation programs as a response to gaps in the broader healthcare system. Specifically, they described their program as responding to known and urgent client needs that were not being adequately addressed by existing healthcare programs and services, including primary care. As one participant said:

*“The second part is looking at a healthcare system level. [Patient navigation] was identified as a real gap in care. You know, we have acute care, we have primary care, but we don’t… it’s almost like we fill a gap for transition care. Uh, and that transition care means that people don’t have to use the emergency room as a default when they feel that their disease is not optimally managed”.* [Participant 7]

Developing and maintaining strong relationships with clients allowed programs to better understand client and community needs, and tailor their services to clients and communities. Participants felt these relationships enabled ongoing implementation and sustainment. As one participant said, “*Our biggest success, is to really listen to our clients and learn from them”* [Participant 11].

*Strong inter-organizational relationships facilitate implementation.* Participants also discussed the importance of strong relationships with health system and community-based programs and services. They felt the development of these inter-organizational relationships facilitated program implementation as navigation programs and enabled them to effectively deliver their services.

*“I think that the connection to community support this is pretty significant … We, the volunteers, are all trained on different community agencies in the area providing services and we give each volunteer like a booklet of what's available in the community. And as they’re meeting with older adults, if something comes up in conversation that, you know, like that might be of interest [to] the person, that there is a service out there that might be able to assist with something, they're trained to sort of provide that information and not just to provide the information but also support a connection to that service.”* [Participant 4]

At the same time, participants from many programs, both health system and community-based, described poor connections to primary care and issues navigating local inter-organizational politics and turf struggles. Participants described the importance of navigating working alongside healthcare providers to fill gaps, while not overstepping within roles or duplicating work that is already being done.

*I didn’t say a lot about primary care because I think primary care is a relationship that needs to be better integrated. And I think part of that is helping to support, but not take away the primary care role. Um, and that is really just more of an education piece but it’s lower to some which is interesting, because people can take away a lot of work, um, that they perhaps can’t provide just by virtue of very busy practices. So for instance, advance care planning facilitation—that takes a lot of time* [Participant 7]

*Limited awareness of program impedes implementation.* Participants from many programs felt there was not enough awareness of their program by healthcare providers, particularly for community-based organizations. This lack of awareness hindered referrals to the program and threatened ongoing implementation and scale-up efforts. As described by one participant:

*“I think some of the barriers were actually um, hospitals are vast, and it was a very small program so constantly communicating with hospital staff, making them aware of our presence so that they could send us referrals as well, that was a challenge for a while because we were continually, continually connecting with hospital social workers letting them know that we are available. So, because things were a little slow in the beginning getting referrals from the hospital or making that connection, we actually took referrals basically from the community”* [Participant 17]

### Inner setting

Participants described three key determinants at the organizational (or inner setting) level: leadership support, the degree of ‘fit’ between staff and the program, and available resources.

*Leadership support and engagement facilitate implementation.* First, participants described the presence of strong leaders who are committed to the program, and who support agency staff by allowing them to try new approaches, encouraging self-care, and offering resources and education to deal with complex client needs. One participant described the program’s leadership in this way:

*“I think if we were bound by policy and procedures to the letter of the law, we would not be able to help as many families as we do. And that is, like I said, one of the beautiful things about working for the organization – that they understand the importance of our job.”* [Participant 10]

*Fit between staff values and skills and the program’s philosophy facilitates implementation*. Participants emphasized that hiring and recruiting the right people are critical to successful implementation and continued impact. In particular, many described the need to hire staff whose personal values and working styles *fit* with the program’s philosophy and the carefulness that is taken by the program to ensure the right people are found. Participants explained the importance of hiring/recruiting those with the necessary skillsets, who believe in and value the program’s philosophy, and who embrace a teamwork approach. As one participant described:

*“I think facilitators are again just the team that we have. There’s a very strict selection process for the people who join the team. It’s certainly not for everybody. It’s a really different way of practicing. And so I think that really helps to facilitate the success of the program.”* [Participant 8]

*Resource availability (or lack thereof) facilitate or impede implementation.* Participants discussed how the availability of resources facilitated or impeded implementation. One frequently discussed resource was training and the capacity to provide staff with the necessary training and skills to care for clients with unique, complex needs. This was viewed as a facilitator to implementation. Participants also described the importance of infrastructure within their programs and organizations to enable efficient operations and to collect data to monitor program effectiveness. Many described limited or inadequate infrastructure, which was a barrier to fully implementing and scaling their programs. As one participant described:

*“I think one of the challenges that we’re having is around the infrastructure that’s needed to comb through the data that we have and really kind of bring out that kind of evaluation through the data. And right now, we don’t have the resources to do that.”* [Participant 12]

### Intervention characteristics

*Ability to adapt program components and delivery facilitates implementation*. At the level of the intervention (i.e., the navigation program itself), participants clearly described the adaptability of their programs, and the ability to evolve as needs change, as a key facilitator to implementation and the effective delivery of services. This responsiveness improved their ability to be patient-centered, as the programs could be modified to meet the complex needs of each client and to changing contexts (i.e., level of funding, community needs).

*“At this point, all we can say is that we think all components of the system that we’ve created are necessary to achieve this impact. I mean – the system was a co-design, co-created system with the community, so that it is an evolving system. We continually, um, adapt and refine it as we discover new ways to improve the delivery. So it’s not, um, it’s not a first garage intervention where every adopter has to do exactly the same thing. Um, again, it’s more of a framework for how communities work together to achieve a quality of life impact by leveraging both informal and formal resources, so that there’s a high degree of flexibility around how it is delivered.”* [Participant 18]

*Inadequate funding to deliver the program impedes implementation*. Participants from most programs emphasized cost considerations as a barrier to full implementation and scale-up. That is, limited and uncertain funding arrangements clearly impeded their efforts to realize widespread implementation and expansion, and sustainment. Indeed, many of the programs operated with much uncertainty from a funding perspective. One participant described it this way:

“When we get a little pocket of money that’s extra and not needed for operational costs, then we purchase some equipment, as we are able to do. But certainly, with the number of clients that we have, we can’t raise enough money from that group of people to keep us sustainable. It’s just not possible. So really, financial sustainability is the biggest challenge that we have.” [Participant 11]

“Barriers I think are resources, or lack thereof essentially. I mean I think we’ve done quite well and we keep getting support, and it’s great. But we’re trying to expand, and we have expanded into other areas of the province. But it’s difficult and it’s limited in terms of funding and uptake and all that kind of stuff” [Participant 8]

### Process of implementation

Participants identified two process-oriented determinants as being particularly germane to implementation and scale-up: champions and evaluation.

*Champions facilitate implementation.* Participants discussed the importance of supportive champions (or, in one program, the lack of champions) to successful implementation. They felt the championing of the program was critical to obtaining initial buy-in, securing initial funding and other resources, maintaining these resources, and building the necessary relationships to continue to deliver personalized care for their clients. These champions existed at the frontline and leadership levels. As one participant stated:

*“We also had champions from executive who would support it and more importantly, three very strong champions who with very limited funding, just persevered in having the program implemented. … That’s definitely a facilitator, um, as you have people who are champions and they do it because they think it needs to be done.”* [Participant 7]

*Evaluation facilitates implementation, refinement, and scale-up.* Many participants described the importance of being able to evaluate the programs to both assess and demonstrate impact. Specifically, participants discussed how conducting evaluations allowed their programs to expand, garner ongoing or additional support, and adapt and refine their services to better align with organizational and/or client needs. Regarding the importance of evaluations, one participant stated:

*I think the other facilitator was that we had good evidence to show that it worked. Um, so that we had data to say this is ...that were having a positive impact because we did do surveys and they did do, um, have data on emergency room visits decreasing, and improvements in the number of patients who had advance care planning completion, and patient satisfaction surveys. So I think the evidence continued to support why this should be implemented* [Participant 7]

### Characteristics of individuals

*Team members’ knowledge and attributes facilitate implementation.* Participants emphasized the importance of having team members who understand their role in the program as well as how the program fits into the larger organizational (or health system) structure. Participants also described the necessity of having program staff whose personal values are highly aligned with community- and home-based care, team-based care, and a palliative approach to care. Upon describing this aspect, one participant said:

*“[We need] someone who’s compassionate. Someone who’s used to working in the community actually visiting people in the community, who feels safe doing that or who knows what to expect in that kind of role definitely. I think those all those things are important.”* [Participant 17]

The participants also spoke about the importance of having staff and volunteers who are integrated into their communities and therefore are aware of available supports and resources, and who have the experience and skills necessary to work with complex clients. As one participant discussed:

*“Volunteers that are connected with hospice, they go through a huge training so they have, they have a lot of knowledge. Many, many of our volunteers are actually retired health care providers … the volunteers can actually support [clients] in a way that many other entities cannot and be aware. I think this is the big difference; like a hospice volunteer is aware of all these needs. Like, spiritual care needs, like social needs, those needs that no one else looks at like they will pay attention. The health care provider will pay attention to the pain, will pay attention to whatever is happening and not about these other parts that our volunteer could probably be a good helper in identifying and in connecting with the resources in the community.”* [Participant 2]

## Discussion

To our knowledge, this was the first Canadian study to explore how navigation programs that support clients with advanced life-limiting illness are successfully implemented within the existing Canadian healthcare system. We found that certain conditions may be important to realizing the successful implementation of these programs. These include addressing and targeting known gaps in client care and establishing strong relationships with other health system and community-based programs, healthcare providers, champions, and clients themselves. Additionally, these programs must carefully select staff or volunteers who align with their values and provide them with the thorough training and support needed to fulfill their role. Furthermore, evaluating and adapting the programs to meet client needs contributes to effective implementation and scale-up. Barriers to implementation most commonly included a lack of resources, including funding. This was heightened by a lack of awareness of the programs from health care providers or other community organizations, which hindered referrals and scalability. Additionally, navigation programs may face challenges when attempting to establish relationships with primary care providers, which limits their coordination with and integration into the healthcare system.

Many of the participants discussed poor relationships with primary care and other healthcare providers due to a lack of awareness or unclear boundaries in terms of roles and responsibilities. Little is known about physician perspectives or knowledge about community-based navigation programs, especially those that support clients as they near EOL. An Ontario study discovered that less than half of physicians surveyed were aware of the majority of community resources available to their patients, particularly those who do not practice within team-based models [38]. Moreover, a recent pan-Canadian initiative found limited integration between primary care and many prioritized services (e.g., palliative care) for community-dwelling older adults experiencing declining function and health [39]. As noted by Cent and Shivers, physicians may struggle to collaborate due to time or resource constraints, which prevent them from learning about these types of programs and how they can assist their clients [40]. However, when physicians work closely with multidisciplinary teams that include patient navigators, studies have demonstrated improved outcomes, such as reduced time to diagnosis or treatment [40]. Although not reported here, our study suggests that improving health care provider and administrative staff knowledge of these services and encouraging collaboration can lead to more effective integration within the healthcare system, which should increase program referrals and ultimately improve client outcomes and improve opportunities for sustainability.

In terms of facilitating implementation, our study resonates with similar findings in other countries. For instance, a scoping review that mainly focused on patient navigation programs in the United States found that these interventions must exist to fill a known gap within the healthcare system and to meet the needs of the patients and community [36]. Furthermore, strong evaluation of these programs is recommended to show that they are filling this gap, while also demonstrating efficacy and cost-effectiveness [36, 41]. However, participants in our study felt that the limited infrastructure hinders many programs’ ability to perform evaluations as they lack the necessary resources to collect and analyze data. Whitley et al. emphasize that such data are crucial to enabling sustainability and garnering support from granting agencies [41]. This funding support is necessary for all aspects of these programs, including staffing, equipment, and patient supports.

Our study highlights the importance of hiring the right staff, or partnering with strong volunteer organizations, to ensure programs comprise team members who share their values, have complementary skillsets, and ideally have experience in EOL care. Kokorealias et al. expand on this in their scoping review of patient navigation programs, where they explain that navigator qualifications may range from those with lived experience to licensed health care providers [42]. They found that most importantly, the navigators must be skilled to support an array of needs, ranging from diagnosis to post-service discharge support, and ongoing health education. The services must be flexible to tailor to the needs of each individual client. Rocque et al. also suggest that training for navigation programs specifically geared towards EOL care should address sensitive topics such as fear of dying and gauging comfort with end-of-life conversations [43].

It is important to note that there is some overlap between the Inner Setting and Characteristics of Individuals domains within the original CFIR framework, which was used to guide this analysis. As a result, the importance of staff/volunteers having the ‘right’ values and skills is presented in both domains. In the Inner Setting domain, we focused on the importance of hiring practices at the organizational level to ensure the staff and volunteers are a good fit. In the Characteristics of the Individuals domain, we emphasized the importance of personal traits (e.g., values, motivations, working styles) and how staff/volunteers use their experiences and skills to appropriately meet the needs of the clients in order for the programs to work and be sustainable. Since the completion of this analysis, an updated CFIR framework was released [44]. This update has revised the Characteristics of Individuals domain, with refined emphasis on clarifying the roles of people involved as well as how staff capabilities, opportunities, and motivation influence implementation and sustainability. The Inner Setting “culture” sub-construct was revised to describe how patient and staff centeredness and learning influence implementation.

This study has numerous practice implications, providing insight into the facilitators and barriers that must be considered when implementing navigation programs for those approaching EOL. First, it highlights the importance of hiring staff or volunteers who share the program’s core values and providing them with comprehensive training to meet the diverse needs of complex clients. Additionally, the study reveals that suboptimal relationships with primary care presents significant challenges to implementation and scale-up efforts, as it hinders referrals and access to patients in need. As a result, future implementation studies should focus on improving these relationships with primary care by increasing healthcare provider and administrative staff knowledge of the services available to their clients, and ensuring roles and responsibilities are well-defined. This study also highlights the importance of strong evaluation efforts to demonstrate program success and sustainability, which can increase buy-in from supportive champions, granting agencies, and other healthcare providers and services. Evaluation should be built into these programs from their conception. For many community-based programs, this may prove challenging as they may not have the resources or expertise to develop and carry-out program evaluation efforts. However, partnering with academic programs or evaluation societies may help support ongoing evaluation processes.

Despite these implications, there are some study limitations that need to be considered. First, the recruitment for this study began at the onset of the COVID-19 pandemic, which meant there was additional stress on these programs and therefore it was challenging to recruit participants. Second, we were also unable to recruit client participants, as the program leaders and staff did not have capacity during the pandemic to assist with recruitment. Third, outside of participants’ experiences, we have no measures of implementation success. Nonetheless, the objective of this study was to understand the facilitators and barriers to implementing these programs into practice; arguably, program staff and leadership have insight into this issue that clients may not have. It is also important to acknowledge that the pandemic may have altered the context in which these programs operate, meaning we must exercise caution when applying these findings today.

## Conclusion

The successful implementation navigation programs into practice appears to benefit from several conditions, including the right staff mix and strong relationships with clients, other community-based organizations, and the healthcare system. Moreover, having strong champions, evaluation capacity, and the ability to adapt to changing circumstances were believed to enable implementation and subsequent scale-up. A significant barrier to program implementation and scale-up is a lack of resources, including funding. Addressing this challenge is critical to ensuring that navigation programs can continue to provide support to those who need it.

### Supplementary Information

**Additional file 1.** Draft Interview Guide – Stakeholder / Decision / Policy Makers.

**Additional file 2. **COREQ statement.

## Data Availability

The datasets generated and/or analysed during the current study are not publicly available due given the qualitative nature of these data, but are available from the corresponding author on reasonable request.

## Abbreviations

CFIR: Consolidated Framework for Implementation Research
EOL: End of Life

## References

[1] Statistics Canada. Canada’s population estimates: Age and sex, July 1, 2021. Statistics Canada; 2021. Available from: https://www150-statcan-gc-ca.ezproxy.library.dal.ca/n1/daily-quotidien/210929/dq210929d-eng.htm.

[2] Public Health Agency of Canada. How Healthy are Canadians? A trend analysis of the health of Canadians from a healthy living and chronic disease perspective. Public Health Agency of Canada; 2017. Available from: https://www.canada.ca/en/public-health/services/publications/healthy-living/how-healthy-canadians.html#s3-3.

[3] Ministry of Health and Long-Term Care. Patients First: Action Plan for Health Care Message from the Minister of Health and Long-Term Care. ON; 2015. Available from: http://www.ontario.ca/health.

[4] The British Columbia Patient-Centred Care Framework. BC; 2015. Available from: http://www.health.gov.bc.ca/library/publications/year/2015_a/pt-centred-care-framework.pdf.

[5] Duncan D. Patient First Review. Saskatchewan Health Initiatives. SK; 2015. Available from: https://www.saskatchewan.ca/government/health-care-administration-and-provider-resources/saskatchewan-health-initiatives/patient-first-review.

[6] Alberta Health Services. Patient First Strategy. Available from: https://www.albertahealthservices.ca/info/Page11981.aspx.

[7] Burge F, Lawson B, Johnston G, Asada Y, McIntyre PF, Flowerdew G (2015). Preferred and actual location of death: what factors enable a preferred home death?. J Palliat Med.

[8] Higginson IJ, Sen-Gupta GJ (2000). Place of care in advanced cancer: a qualitative systematic literature review of patient preferences. J Palliat Med.

[9] Gomes B, Calanzani N, Gysels M, Hall S, Higginson IJ (2013). Heterogeneity and changes in preferences for dying at home: a systematic review. BMC Palliat Care.

[10] Burge F, Lawson B, Johnston G, Asada Y, McIntyre PF, Grunfeld E (2014). Bereaved family member perceptions of patient-focused family-centred care during the last 30 days of life using a mortality follow-back survey: does location matter?. BMC Palliat Care.

[11] Morrison RS (2018). A national palliative care strategy for Canada. J Palliat Med.

[12] Saint Elizabeth. Highlights from the Environics End-of-Life Care Survey for Saint Elizabeth. Markham, ON: Saint Elizabeth; 2013. Available from: https://sehc.com/SEHC/media/foundation/pdfs/End-of-life-survey-fact-sheet-FINAL.pdf.

[13] Aubin M, Giguère A, Martin M, Verreault R, Fitch MI, Kazanjian A (2012). Interventions to improve continuity of care in the follow-up of patients with cancer. Cochrane Database Syst Rev.

[14] Marshall D, Howell D, Brazil K, Howard M, Taniguchi A (2008). Enhancing family physician capacity to deliver quality palliative home care: an end-of-life, shared-care model. Can Fam Physician.

[15] Seow H, Brazil K, Sussman J, Pereira J, Marshall D, Austin PC (2014). Impact of community based, specialist palliative care teams on hospitalisations and emergency department visits late in life and hospital deaths: a pooled analysis. BMJ.

[16] Katz A, Martens P, Chateau D, Bogdanovic B, Koseva I (2014). Do primary care physicians coordinate ambulatory care for chronic disease patients in Canada?. BMC Fam Pract.

[17] Di Palo KE, Patel K, Assafin M, Piña IL (2017). Implementation of a patient navigator program to reduce 30-day heart failure readmission rate. Prog Cardiovasc Dis.

[18] Percac-Lima S, Ashburner JM, Rigotti NA, Park ER, Chang Y, Kuchukhidze S (2018). Patient navigation for lung cancer screening among current smokers in community health centers a randomized controlled trial. Cancer Med.

[19] Rodday AM, Parsons SK, Snyder F, Simon MA, Llanos AAM, Warren-Mears V (2015). Impact of patient navigation in eliminating economic disparities in cancer care. Cancer.

[20] Freund KM, Battaglia TA, Calhoun E, Darnell JS, Dudley DJ, Fiscella K (2014). Impact of patient navigation on timely cancer care: the patient navigation research program. J Natl Cancer Inst.

[21] Ko NY, Darnell JS, Calhoun E, Freund KM, Wells KJ, Shapiro CL (2014). Can patient navigation improve receipt of recommended breast cancer care? Evidence from the National patient navigation research program. J Clin Oncol.

[22] Balaban RB, Galbraith AA, Burns ME, Vialle-Valentin CE, Larochelle MR, Ross-Degnan D (2015). A patient navigator intervention to reduce hospital readmissions among high-risk safety-net patients: a randomized controlled trial. J Gen Intern Med.

[23] Chan RJ, Milch VE, Crawford-Williams F, Agbejule OA, Joseph R, Johal J (2023). Patient navigation across the cancer care continuum: an overview of systematic reviews and emerging literature. CA.

[24] Pesut B, Hooper B, Jacobsen M, Nielsen B, Falk M, ‘Connor B. Nurse-led navigation to provide early palliative care in rural areas: a pilot study. BMC Palliative Care. 2017 Jun 5;16. 10.1186/s12904-017-0211-2 PMC5460511 28583176

[25] Bainbridge D, Seow H, Sussman J (2016). Common components of efficacious in-home end-of-life care programs: a review of systematic reviews. J Am Geriatr Soc.

[26] Brumley RD, Enguidanos S, Cherin DA (2003). Effectiveness of a home-based palliative care program for end-of-life. J Palliat Med.

[27] Pesut B, Duggleby W, Warner G, Fassbender K, Antifeau E, Hooper B (2017). Volunteer navigation partnerships: piloting a compassionate community approach to early palliative care. BMC Palliat Care.

[28] Dolovich L, Oliver D, Lamarche L, Agarwal G, Carr T, Chan D (2016). A protocol for a pragmatic randomized controlled trial using the health teams advancing patient experience: strengthening quality (Health TAPESTRY) platform approach to promote person-focused primary healthcare for older adults. Implement Sci.

[29] Anthonisen G, Luke A, MacNeill L, MacNeill AL, Goudreau A, Doucet S (2023). Patient navigation programs for people with dementia, their caregivers, and members of the care team: a scoping review. JBI Evid Synth.

[30] Pratt-Chapman ML, Silber R, Tang J, Le PTD (2021). Implementation factors for patient navigation program success: a qualitative study. Implement Sci Commun.

[31] Urquhart R, Kendell C, Pfaff K, Stajduhar K, Patrick L, Dujela C (2023). How do navigation programs address the needs of those living in the community with advanced, life-limiting Illness? A realist evaluation of programs in Canada. BMC Palliat Care.

[32] Ray Pawson, Nicholas Tilley. Realistic Evaluation. SAGE Publications Ltd; 1997. Available from: https://uk.sagepub.com/en-gb/eur/realistic-evaluation/book205276. Cited 2023 Jan 20.

[33] Damschroder LJ, Aron DC, Keith RE, Kirsh SR, Alexander JA, Lowery JC (2009). Fostering implementation of health services research findings into practice: a consolidated framework for advancing implementation science. Implement Sci.

[34] DeLurio J, Erinoff E, Hulshizer R, Robertson D, Wilkinson B, Schoelles K. Horizon Scanning Protocol and Operations Manual September 2015 Revision. Rockville, MD: Agency for Healthcare Research and Quality; 2015.

[35] Touzel M, Shadd J (2018). Content validity of a conceptual model of a palliative approach. J Palliat Med.

[36] Valaitis RK, Carter N, Lam A, Nicholl J, Feather J, Cleghorn L (2017). Implementation and maintenance of patient navigation programs linking primary care with community-based health and social services: a scoping literature review. BMC Health Serv Res.

[37] Sandelowski M (2000). Whatever happened to qualitative description?. Res Nurs Health.

[38] Kiran T, Rodrigues J, Aratangy T, Devotta K, Sava N, O’Campo P (2020). Awareness and use of community services among primary care physicians. Healthcare policy = Politiques de sante.

[39] Stewart T, Dionne É, Urquhart R, Oelke ND, Couturier Y, Haggerty CMS and J. Integrating Health and Social Care for Community-Dwelling Older Adults: A Description of 16 Canadian Programs. Healthcare Policy. 2023 Oct 5;19(Special Issue). Available from: https://www.longwoods.com/content/27177/healthcare-policy/integrating-health-and-social-care-for-community-dwelling-older-adults-a-description-of-16-canadian. Cited 2023 Nov 28. 10.12927/hcpol.2023.27177 PMC10594941 37850707

[40] Cent MA, Shivers K (2010). Physician collaboration & quality improvement outcomes: through the establishment of disease-site teams. Oncology Issues.

[41] Whitley E, Valverde P, Wells K, Williams L, Teschner T, Shih YCT (2011). Establishing common cost measures to evaluate the economic value of patient navigation programs. Cancer.

[42] Kokorelias KM, Shiers-Hanley JE, Rios J, Knoepfli A, Hitzig SL (2021). Factors Influencing the implementation of patient navigation programs for adults with complex needs: a scoping review of the literature. Health Serv Insights.

[43] Rocque GB, Dionne-Odom JN, Sylvia Huang CH, Niranjan SJ, Williams CP, Jackson BE (2017). Implementation and impact of patient lay navigator-led advance care planning conversations. J Pain Symptom Manage.

[44] Damschroder LJ, Reardon CM, Widerquist MAO, Lowery J (2022). The updated consolidated framework for implementation research based on user feedback. Implement Sci.

