# Supplementary material for: Navigation programs to support community-dwelling individuals with life-limiting illness: determinants of implementation
Source: BMC Health Serv Res. 2024 Jan 6;24:39. doi: 10.1186/s12913-024-10541-y (PMC10770879; doi:10.1186/s12913-024-10541-y)
Supplement: Supplementary file 2 — Additional file 2. COREQ statement. [file 12913_2024_10541_MOESM2_ESM.docx]

**Additional File 2**

COREQ statement

This document reports key study aspects according to the COREQ guidelines for reporting qualitative research.

Consolidated criteria for reporting qualitative studies (COREQ): 32-item checklist

| **No** | **Item** | **Guide questions/description** | **Author response** |
| --- | --- | --- | --- |
| **Domain 1: Research team and reflexivity** |  |  |  |
| Personal Characteristics |  |  |  |
| 1. | Interviewer/facilitator | Which author/s conducted the interview or focus group? | CK, FF, or CD; stated in text (methods section, pg 7) |
| 2. | Credentials | What were the researcher's credentials? *E.g. PhD, MD* | GW, KP, KS, LP, and RU have PhDs and are experienced qualitative researchers with >15 years of experience each; SS was a Master’s student, trained in qualitative methods by RU; CK and FF were PhD students with expertise in qualitative methods; CD was a longstanding research staff, with experience in qualitative methods. |
| 3. | Occupation | What was their occupation at the time of the study? | GW, KP, KS, LP, RU: All faculty members at their respective universities.  CK: PhD student, research coordinator  CK, FF: PhD student, research coordinator  SS: Master’s student, research associate  CD: Research coordinator |
| 4. | Gender | Was the researcher male or female? | All female |
| 5. | Experience and training | What experience or training did the researcher have? | See above answers. All researchers had experience in qualitative methods. |
| Relationship with participants |  |  |  |
| 6. | Relationship established | Was a relationship established prior to study commencement? | There was no relationship between the interviewer [CK, FF, CD] and any participants prior to study commencement. |
| 7. | Participant knowledge of the interviewer | What did the participants know about the researcher? e*.g. personal goals, reasons for doing the research* | The participants would not have known anything about the interviewer prior to the study; some participants may have known who the lead PI/co-PIs were before study commencement due to the nature of their research. All would have understood the reasons for doing the research as part of the informed consent process. |
| 8. | Interviewer characteristics | What characteristics were reported about the interviewer/facilitator? e.g. *Bias, assumptions, reasons and interests in the research topic* | No characteristics are reported about the interviewer. The interviewers were PhD students or research staff with experience in qualitative methods. |
| **Domain 2: study design** |  |  |  |
| Theoretical framework |  |  |  |
| 9. | Methodological orientation and Theory | What methodological orientation was stated to underpin the study? *e.g. grounded theory, discourse analysis, ethnography, phenomenology, content analysis* | The overall study used realist evaluation as its underpinning orientation. The analysis for this component of the study (implementation) used techniques in qualitative description, following the approach of Sandelowski (2000). These underpinnings are cited in the manuscript on page 6 and 8. |
| Participant selection |  |  |  |
| 10. | Sampling | How were participants selected? *e.g. purposive, convenience, consecutive, snowball* | Purposive sampling. Criteria to identify participants are described in text and criteria to describe programs are described in Box 1. |
| 11. | Method of approach | How were participants approached? e*.g. face-to-face, telephone, mail, email* | Email; stated in text (methods section, pg 7) |
| 12. | Sample size | How many participants were in the study? | 12; stated in text (results section, pg 8) |
| 13. | Non-participation | How many people refused to participate or dropped out? Reasons? | Only one person agreed to be interviewed, but did not participate (due to workload issues during the pandemic). |
| Setting |  |  |  |
| 14. | Setting of data collection | Where was the data collected? e*.g. home, clinic, workplace* | Interviews were conducted in person, or via telephone or videoconferencing, depending on the participant’s preference and geographic location. |
| 15. | Presence of non-participants | Was anyone else present besides the participants and researchers? | No. |
| 16. | Description of sample | What are the important characteristics of the sample? *e.g. demographic data, date* | Detailed demographic data are not presented, given we did collect detailed (participant-level) data due to the nature of the study (focus on implementation of programs). We also have not provided detailed data on each program, because we fear these data would compromise participant confidentiality. |
| Data collection |  |  |  |
| 17. | Interview guide | Were questions, prompts, guides provided by the authors? Was it pilot tested? | The interview guide is available as a supplemental file. Interview questions and prompts were crafted by RU, GW, KS, KP, and CK. All have experience with methods (qualitative) and substantive (navigation, palliative care) areas. The interview guide was not pilot tested. However, per a realist approach, interview guides were adapted to explore and expand on salient theoretical constructs as the program theory was being developed. Interview questions were also adapted as needed based on the person being interviewed and his/her role in the program. |
| 18. | Repeat interviews | Were repeat interviews carried out? If yes, how many? | No; stated in text (methods section, pg 7). |
| 19. | Audio/visual recording | Did the research use audio or visual recording to collect the data? | Yes, audio recording; stated in text (methods section, pg 8). |
| 20. | Field notes | Were field notes made during and/or after the interview or focus group? | No. |
| 21. | Duration | What was the duration of the interviews or focus group? | Interview duration ranged from 24m56s to 1h28m40s; stated in text (methods section, pg 7). |
| 22. | Data saturation | Was data saturation discussed? | We heard very similar concepts across our entire dataset, despite differences in program settings and funding mechanisms. However, as stated in our Discussion section, we were also unable to recruit client participants. We may have gained additional perspectives and new concepts if we were able to interview clients of these programs. |
| 23. | Transcripts returned | Were transcripts returned to participants for comment and/or correction? | No. |
| **Domain 3: analysis and findings** |  |  |  |
| Data analysis |  |  |  |
| 24. | Number of data coders | How many data coders coded the data? | All researchers read and re-read the transcripts, and developed initial program theories, as aligned with realist evaluation. One researcher [SS] then coded all the transcripts. This coding was reviewed by three authors (RU, GW, CK), all familiar with the dataset. The entire team then reviewed, questioned, and refined the themes; stated in text (methods section, pg 8). |
| 25. | Description of the coding tree | Did authors provide a description of the coding tree? | No. |
| 26. | Derivation of themes | Were themes identified in advance or derived from the data? | Derived from the data but organized and presented according to the five CFIR domains (methods section, pg 8). |
| 27. | Software | What software, if applicable, was used to manage the data? | NVIVO 12 was used to manage the coding process; stated in text (methods section, pg 8). |
| 28. | Participant checking | Did participants provide feedback on the findings? | No. |
| Reporting |  |  |  |
| 29. | Quotations presented | Were participant quotations presented to illustrate the themes / findings? Was each quotation identified? e*.g. participant number* | Yes. |
| 30. | Data and findings consistent | Was there consistency between the data presented and the findings? | Yes. |
| 31. | Clarity of major themes | Were major themes clearly presented in the findings? | Yes. |
| 32. | Clarity of minor themes | Is there a description of diverse cases or discussion of minor themes? | The text describes the nuances and differences across programs. |
